# Systematic review of CYFRA 21-1 as a prognostic indicator and its predictive correlation with clinicopathological features in Non-small Cell Lung Cancer: A meta-analysis

**DOI:** 10.18632/oncotarget.14022

**Published:** 2016-12-19

**Authors:** Zipu Yu, Guofei Zhang, Maoying Yang, Sai Zhang, Baiqin Zhao, Gang Shen, Ying Chai

**Affiliations:** ^1^ Department of Thoracic Surgery, 2nd Affiliated Hospital, Zhejiang University, Hangzhou, China

**Keywords:** Cytokeratin 19 fragment (CYFRA 21-1), Non-small Cell Lung Cancer(NSCLC), 2-year overall survival, clinicopathological features

## Abstract

**AIM:**

To evaluate the value of Cytokeratin 19 fragment for its survival prognostic indicator and predictive correlation with clinicopathological features in Non-small Cell Lung Cancer.

**METHODS:**

Eligible studies or databases for articles were retrieved via search systematically. Pooled effect was calculated to evaluate the association between Cytokeratin 19 fragment level and long-term overall survival, as well as the tumor clinicopathological features in Non-small Cell Lung Cancer patients. A fixed-effects or random-effects model was used to calculate the Pooled risk ratios (RRs) and corresponding 95 % confidence intervals (CIs).

**RESULTS:**

Six studies were up to the selection criteria. This meta-analysis indicated that Cytokeratin 19 fragment high level expression correlated with lower 2-year overall survival (RR =0.47; 95%CI: 0.28-0.79), higher Tumor Node Metastasis stage (II+III+IV) (RR =1.43; 95%CI: 1.15-1.76) in Non-small Cell Lung Cancer. The pooled RR estimates indicated that there is no statistical significance of Cytokeratin 19 fragment level expression in the advanced Non-small Cell Lung Cancer (IIIB+IV) (RR =1.43, 95% CI: 0.85-2.43).

**CONCLUSION:**

Cytokeratin 19 fragment is a negative prognosis indicator and its high level expression indicates higher Tumor Node Metastasis pathological stage (II+III+IV) in Non-small Cell Lung Cancer. In advanced Non-small Cell Lung Cancer, the level of serum Cytokeratin 19 fragment appears to provide more prognostic information than it does for clinical Tumor Node Metastasis stage information. Further studies are required to confirm our results.

## INTRODUCTION

Lung cancer is becoming the leading cause of cancer death worldwide, with most patients dying of tumour recurrence and metastasis [1]. Non-small cell lung cancer (NSCLC) accounts for approximately 85% of all lung cancer cases [2]. In terms of both frequency and clinical significance, squamous cell carcinoma, small cell carcinoma, adenocarcinoma and large cell carcinoma are most important histologic categories. Lung cancer is known to be a highly treatment-refractory cancer. Treatment of non-small-cell lung cancer (NSCLC) is probably one of the great challenges of medical treatment owing to an increasing incidence and poor prognosis [3]. Difficulties in establishing treatment guidelines exist owing to considerable heterogeneous clinical behaviours of NCSLC. Stage of the disease is the strongest prognostic indicator. Nevertheless, other prognostic factors such as tumour histology, lymphatic and blood vessels involvement are described in the literatures [4, 5]. Hitherto, the prognosis was mainly defined by three variables: the stage of the disease, the performance status and different patient conditions including age [6, 7, 8, 9].

To improve the survival of lung cancer patients, early detection and surgical resection is necessary. In the early stage of non-small cell lung cancer (NSCLC), surgical treatment is associated with the best prognosis. Current 5-year survival rates are 50-70% in pathological stage I of the disease [10]. A few options for the detection of NSCLC are available to detect early NSCLC. Screening using sputum cytology, computed tomography (CT) scanning and histological confirmation through biopsy specimens are available method for lung cancer diagnosis, but these methods are of limited use in practice owing to their diagnose accuracy, inconveniences and other disadvantages.

Despite extensive pre-operative evaluation, still a significant proportion of the patients are diagnosed with late pathological stage combined with substantially worse survival. Recently, the use of serum biomarkers to detect cancer in early stages has become an area of interest. These biomarkers were found in the involvement of development and progression of cancer [11], of which cytokines are attracting a great deal of interest as a candidate marker. They have been found to correlate with the clinicopathological features of various lymphoid and non-lymphoid neoplasms [12–19]. Cytokeratin 19 fragment (CYFRA 21-1) is a fragment of cytokeratin (CK) 19. CKs exist in many normal and malignant epithelial cells and they can be detected by a solid-phase enzyme-linked sandwich immunoradiometric assay (IRMA) [20, 21, 22, 23]. CK have been classified into 20 subtypes based on differences in the molecular mass and isoelectric point as determined by 2-dimensional electrophoresis. CK types 1-8 are categorized as type I CKs and CKs 9-20 as type II CKs. Microfilaments are heteropolymers formed from type I and type II keratins, and constitute the cytoskeleton. CK19 is a soluble type I CK, and has the lowest molecular mass (40 kDa) among the CKs. Accelerated CK19 degradation occurs as a result of increased protease activity of caspase-3, C-terminus of cytokeratin 19 (CYFRA 21-1) are released into circulation in malignant tissues [24]. Early clinical studies have shown that elevated levels of serum CYFRA 21-1 were detected in patients with non-small cell lung cancer (NSCLC). But one can observe that there are some uncertainties regarding the exact risk ratio of death associated with a high serum CYFRA 21-1 level by analyzing different studies [25–32].

Given that these studies have been published recently, our aims of the current study are to (1) investigate the exact risk ratio of the long survival associated with a high serum CYFRA 21-1 level for NSCLC, (2) identify relationships between CYFRA 21-1 high level expression and TNM pathological stage.

## RESULTS

### Description of trials

**Figure 1:**
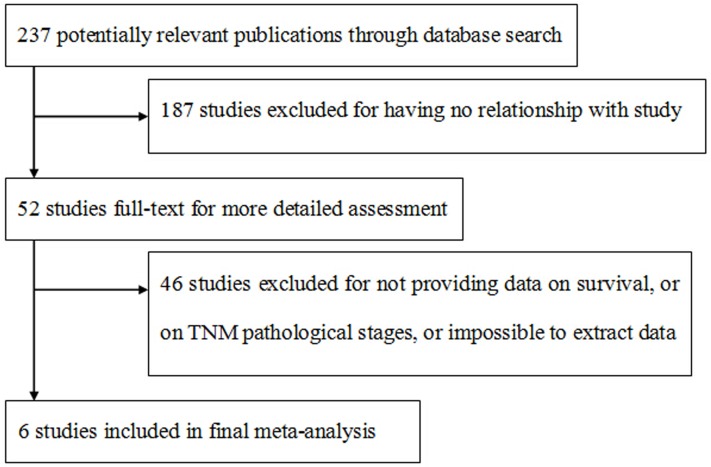
Flow chart indicating the process of selecting articles for meta-analysis

The initial search strategy retrieved identified 237 articles, of which 185 articles were excluded as not being relevant. The remaining studies were assessed for eligibility, finally 6 studies met [31, 33–37] our entry criteria and were retrieved for more detailed evaluation (Figure 1). Their characteristics are summarized in Table 1. Among the 824 patients, 347 patients showed high level expression. Of six included studies, two provided data on 5-year overall survival while the other provided data on 2-year overall survival. The studies were conducted in four countries (Japan, South Korea, USA and Poland).

**Table 1:** Characteristics information of the included studies

Ref	Country of origin	Sample size	Study quality(points)	Survival rate analysis
J. Niklinski, et al [35]	Poland and Italy	94	5/9	Reported
Kosuke Kashiwabara et al[31]	Japan	120	5/9	Reported
Hisashi Suzuki, et al [36]	Japan	101	5/9	Reported
Minkyu Jung, et al [37]	Korea	123	7/9	Reported
Martin J. Edelman, et al [38]	USA	88	5/9	Reported
Seong Yong Park, et al [39]	Korea	298	7/9	Reported

Study quality was listed using the results of the Newcastle -Ottawa questionnaire.

### A prognostic indicator of CYFRA 21-1 for 2-year overall survival

**Figure 2:**
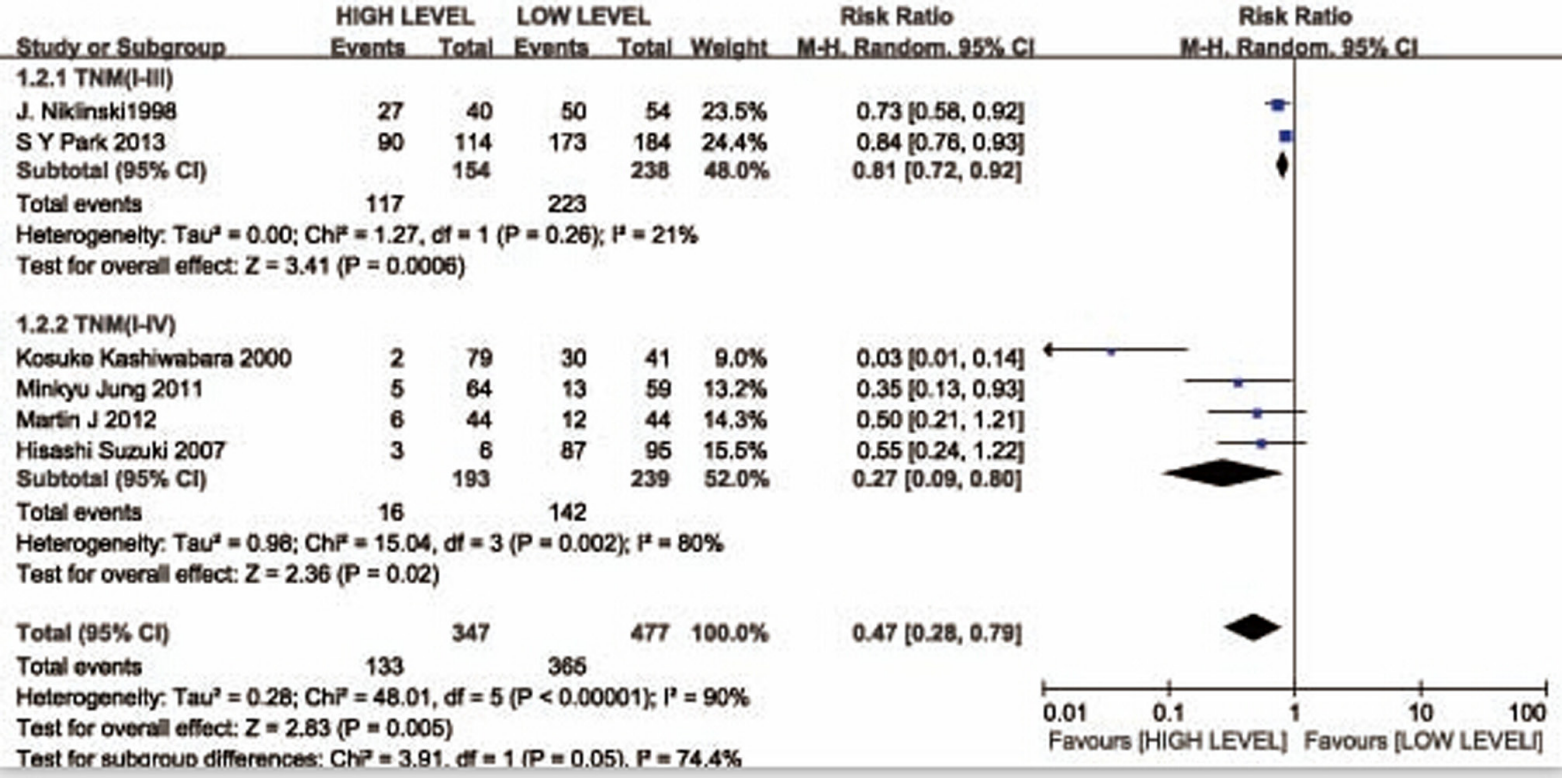
Forest plot presenting 2-year overall survival from the studies included 95% CI: 95% confidence interval.

Owing to the different data of overall survival provided by studies, the 2-year overall survival (long survival) was extracted from six studies. Meta-analysis indicated that patients with CYFRA 21-1 high level expression suffered with a lower 2-year overall survival ((RR =0.47; 95%CI: 0.28-0.79)). The random effects model was used because of the heterogeneity (*I*^2^ = 90.0%) (Figure 2).

### Evaluation of heterogeneity

A subgroup analysis was performed because of the heterogeneity to determine whether different stage TNM of NSCLC would influence the results. The stage TNM of NSCLC in the TNM ( I-III ) groups and TNM( I-IV ) groups had significantly differences (P = 0.05, *I*^2^ = 74.4%) (Figure 2).

### Subgroup analyses

Among the six studies, there was two groups for TNM ( I-III ),of which the risk of 2-year overall survival in the high level group was distinct from that in the low level group (RR = 0.81, 95% CI 0.72–0.92, P = 0.0006; Figure 2). The other four groups were included in the same group for more information of TNM (I-IV) stages, of which the risk of 2-year overall survival in the high level group was lower that in the low level group (RR = 0.27, 95% CI 0.09–0.80, P = 0.005; Figure 2).

### Correlation between CYFRA 21-1 level and the tumor clinicopathological features in NSCSLC patients

**Figure 3:**
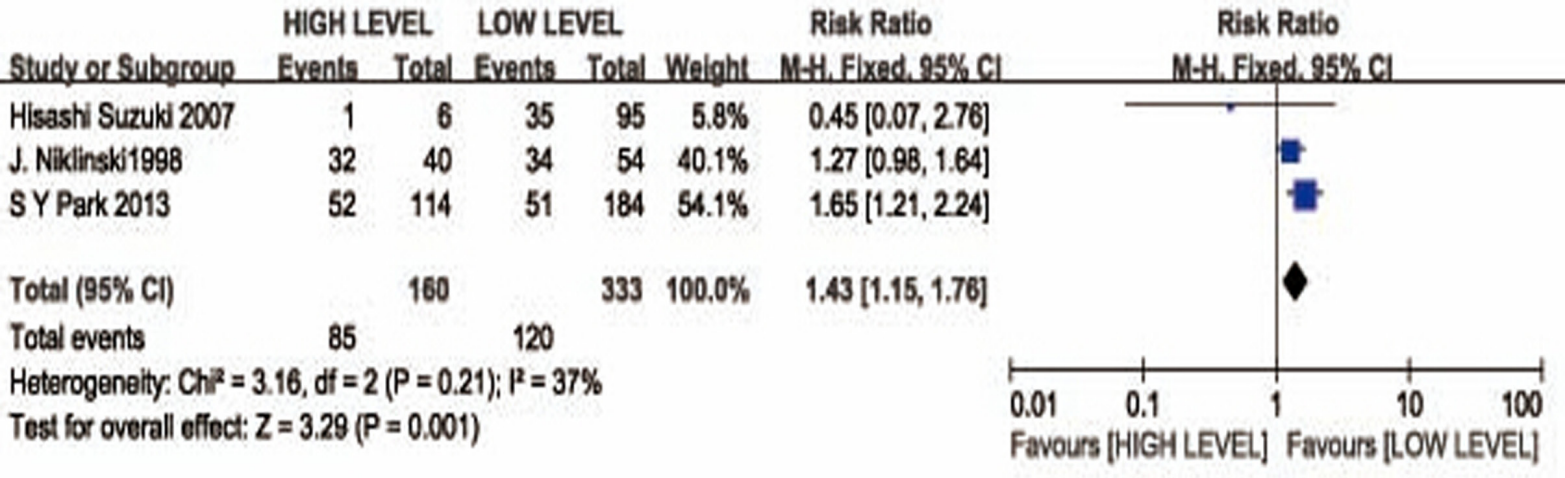
Forest plot presenting TNM(II-IV) from the studies included 95% CI: 95% confidence interval.

**Figure 4:**
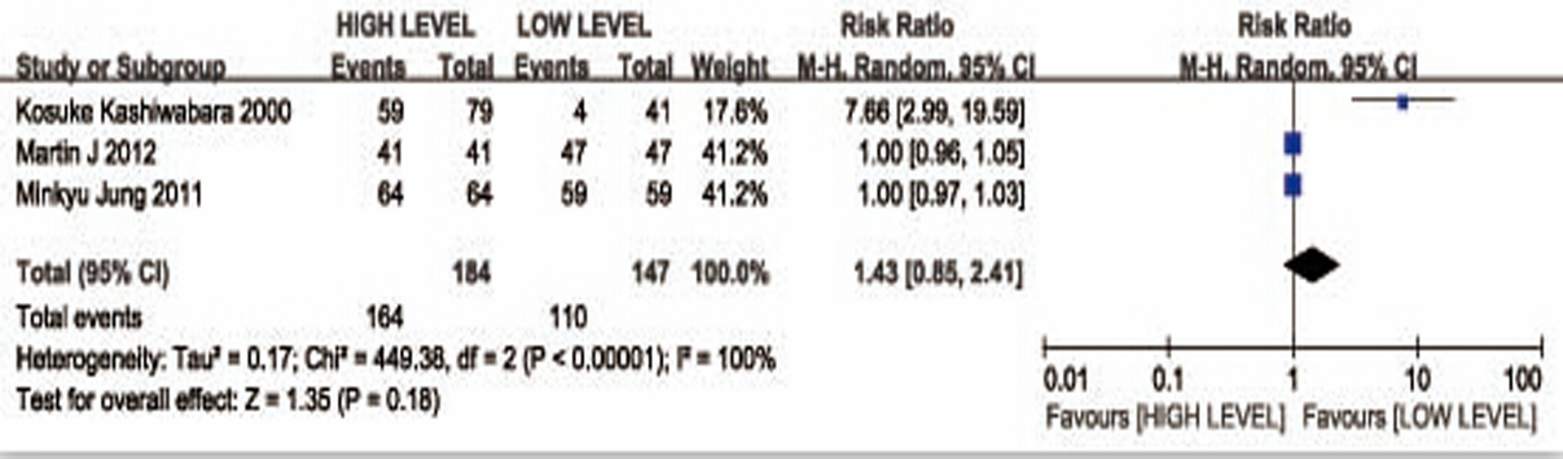
Forest plot presenting TNM(IIIB-IV) from the studies included 95% CI: 95% confidence interval.

Pooled risk ratios (RRs) showed that CYFRA 21-1 high level in NSCSLC patients was associated with biologically higher TNM pathological stage(II + III + IV) (RR= 1,43; 95%CI: 1.15-1.76) (Figure 3). There were three groups including for analyzing relationship of CYFRA 21-1 expression level with the advanced TNM pathological stage(IIIB + IV). As far as the advanced pathological stage of non-small cell lung cancer patients is concerned, the pooled RR estimates indicated that there was no statistical significance of CYFRA 21-1 level expression between high level expression and low level expression (RR =1.43, 95% CI: 0.85-2.41) (Figure 4).

## DISCUSSION

Meta-analysis is considered to be an important tool for randomized controlled trials (RCTs) and non-randomized controlled trials (NRCTs). By summarizing the results of published studies, we aimed to evaluate the relationship existent to provide useful information for clinical decision-making in NSCLC patients. In this systematic review and meta-analysis, we found that CYFRA 21-1 high level expression in NSCLC correlated with lower 2-year overall survival and higher TNM stage(II + III + IV). There is no statistical significance of CYFRA 21-1 level expression between high level expression and low level expression in the advanced pathological stage of non-small cell lung cancer patients (IIIB + IV).

CYFRA 21-1 was identified in 1993 firstly as a valuable marker in lung cancer patients, which could be measured by a sandwich enzyme-linked immunosorbent assay to detect a soluble cytokeratin 19 fragment that is expressed in bronchial epithelium and malignant lung tumors. Serum CYFRA 21-1 level was not influenced by sex and smoking habits [38, 39], which was found to be an independent prognostic factor of survival and tumour relapse [40]. The meta-analysis about prognostic significance of CYFRA 21-1 has been studied in several articles, which have indicated that high level expression of CYFRA 21-1 implies a poor prognosis [32, 34, 41–48]. In this study, we found that the 2-year overall survival in the high level group was significantly lower than that in the low level group group, which is consistant with studies previous.

Additionally, we analyzed the relationship between the expression of CYFRA 21-1 and clinicopathologic features of NSCLC. Studies were excluded if not providing information between CYFRA 21-1 level expression and clinicopathologic features. Our result also demonstrated that CYFRA 21-1 high level expression was correlated with higher TNM stage(II + III + IV). As mentioned previously, Stage of the disease is the strongest prognostic indicator. NSCLC could be devied into four stage in clinical practice, including (I+II+III+IV) stages. StageI is a early- stage NSCLC, which have opportunity for surgical treatment while stages of (IIIB+IV) belongs to the advanced Non–Small-Cell Lung Cancer having a worse prognosis. Baesd on the pathological meaning for clinical TNM practices and information from the studies, we divided studies into two subgroups and made analysis respectively. The analysis indicated that CYFRA 21-1 high level expression in Non-small Cell Lung Cancer correlated with higher TNM stage (II+III+IV). As far as the the advanced Non-small Cell Lung Cancer is concerned, the result indicated that there is no statistical difference of CYFRA 21-1 level expression between high level expression and low level expression. The latest study, consistant with our study for the conclusion about CYFRA 21-1 as a poor prognosis, not analyzed its relationship between the expression of CYFRA 21-1 and clinicopathologic features of NSCLC.[49] This is the original discovery in our meta-analysis.

A meta-analysis cannot solve problems with confounding factors that could be internal to the recruited studies [50]. The results should be interpreted with caution because of several limitations to this meta-analysis. First, reports not in English were excluded. The risk of language bias had to be considered, possible publication bias could emerge as the result of the strategy of selection and the exclusion of studies. Second, the data came from NRCTs. Nevertheless, Study [51] had indicated that a meta-analysis of well designed NRCTs of study were probably as accurate as those carried out on RCTs. Third, there was heterogeneity across studies. A random-effects model was used to take variation into consideration, we controlled influence of the heterogeneity by dividing studies into subgroups for analysis. Fourth, types of methodology for CYFRA 21-1 measurement were not elaborated in studies included. By means of diverse methodology adopted in 6 studies inclued, we divided patients into one group with a CYFRA 21-1 high level expression or the other group with a CYFRA 21-1 low level expression. Finally. Comparing with 2-year overall survival, 5-year overall survival was more frequently used in clinical practice. 2-year overall survival was adopted in our study because of information provided in the studies.

In conclusion, the results of this meta-analysis showed that Cytokeratin 19 fragment (CYFRA 21-1) is a negative prognosis indicator and its high level expression indicates higher Tumor Node Metastasis (TNM) pathological stage (II+III+IV) in Non-small Cell Lung Cancer (NSCLC). The level of serum CYFRA 21-1 appears to provide more prognostic information than it does for clinical TNM stage information in advanced stage Non-small Cell Lung Cancer(IIIB + IV). Further studies are needed to confirm our results.

## MATERIALS AND METHODS

### Study selection

A literature search was conducted using multiple databases including PubMed, the Cochrane Library and the Web of Science to find out studies for all articles published in English before January, 2016. The terms used for thesearch were: “CYFRA 21-1” and “Non-small Cell Lung Cancer”. Reference lists of all retrieved articles were also manually searched for additional studies. Two reviewers extracted the data from each study independently. All relevant text, tables, and figures were reviewed for data extraction, and any disagreement was resolved by consensus.

### Eligibility criteria

The inclusion criteria of this meta-analysis were: (1) patients with NSCLC diagnosed by pathology; (2) studies that examined the relationship between CYFRA 21-1 level expression and the long survival of NSCLC; and (3) studies that identified relationships between CYFRA 21-1 level expression and TNM pathological stage.

The studies or data were also excluded for: (1) studies lacking information on survival; (2) studies lacking information on TNM pathological stage; (3) overlapping articles or duplicate data; (4) articles about animals or conference records; (5) being impossible to extract the appropriate data from the published results. Abstracts, letters, editorials and expert opinions, reviews without original data, case reports, and studies lacking a control group were excluded.

### Outcomes of interest

We mainly aimed at evaluating the prognostic value of CYFRA 21-1 high level expression in NSCLC patients regarding 2-year or 5-year overall survival. Our second aim was to assess the association of CYFRA 21-1 high level expression in NSCLC patients with tumor clinicopathological features, indicated by tumor node metastasis (TNM) stage.

### Data abstraction and quality assessment

Two reviewers independently extracted the following parameters from each study: (1) first author and year of publication; (2) number of population who were included in studies; (3) number of population who had a high or low level expression of CYFRA 21-1; (4) the long-term overall survival (2-year overall survival); and (5) the clinicopathological features. Data abstraction and quality assessment were performed as described previously [52]. Quality assessment was performed with the Newcastle-Ottawa quality assessment scale(NOS). The Newcastle–Ottawa scale assesses the quality of study based on the following three aspects: (i) the selection of the study cohort (or cases/controls), (ii) the comparability of the cohorts (or cases/controls) and (iii) the outcome assessment for a cohort study, or the determination of the exposure for a case–control study. The meta-analysis was performed according to the PRISMA guidelines [53].

### Statistical analysis

Review Manager 5.2(RevMan 5.2®, Nordic Cochrane Center and Copenhagen, Denmark) was used to perform analysis. We analyzed dichotomous variables using estimation of Risk ratios (RR) with 95%CI. The pooled effect was calculated using either a fixed-effects or a random-effects model. The *I*^2^ statistic was used to quantify the statistical heterogeneity of the studies. When the *I*^2^ value was >50%, indicating the presence of variability among the studies, a random-effects models rather than fixed-effects models was used to perform the meta-analysis. Risk ratios (RRs) with a 95% confidence interval (CI) were used to report the differences in clinical outcomes between the high level expression and low level expression of population. Forest plots were used to present the results of this meta-analysis. A *P*-value <0.05 was considered to be significantly different.

## References

[1] Ginsberg MS, Grewal RK, Heelan RT (2007). Lung cancer. Radiologic clinics of North America.

[2] Jemal A, Siegel R, Ward E, Hao Y, Xu J, Murray T, Thun MJ (2008). Cancer statistics, 2008. CA Cancer J Clin.

[3] (1997). Clinical practice guidelines for the treatment of unresectable non-small-cell lung cancer. Adopted on May 16, 1997 by the American Society of Clinical Oncology. Journal of clinical oncology.

[4] Chansky K, Sculier JP, Crowley JJ, Giroux D, Van Meerbeeck J, Goldstraw P (2009). The International Association for the Study of Lung Cancer Staging Project: prognostic factors and pathologic TNM stage in surgically managed non-small cell lung cancer. Journal of thoracic oncology.

[5] Tsuchiya T, Hashizume S, Akamine S, Muraoka M, Honda S, Tsuji K, Urabe S, Hayashi T, Yamasaki N, Nagayasu T (2007). Upstaging by vessel invasion improves the pathology staging system of non-small cell lung cancer. Chest.

[6] Inoue K, Sato M, Fujimura S, Sakurada A, Takahashi S, Usuda K, Kondo T, Tanita T, Handa M, Saito Y, Sagawa M (1998). Prognostic assessment of 1310 patients with non-small-cell lung cancer who underwent complete resection from 1980 to 1993. The Journal of thoracic and cardiovascular surgery.

[7] Andre F, Grunenwald D, Pignon JP, Dujon A, Pujol JL, Brichon PY, Brouchet L, Quoix E, Westeel V, Le Chevalier T (2000). Survival of patients with resected N2 non-small-cell lung cancer: evidence for a subclassification and implications. Journal of clinical oncology.

[8] Firat S, Byhardt RW, Gore E (2002). Comorbidity and Karnofksy performance score are independent prognostic factors in stage III non-small-cell lung cancer: an institutional analysis of patients treated on four RTOG studies. Radiation Therapy Oncology Group. International journal of radiation oncology, biology, physics.

[9] Merrill RM, Henson DE, Barnes M (1999). Conditional survival among patients with carcinoma of the lung. Chest.

[10] Cho S, Song IH, Yang HC, Kim K, Jheon S (2013). Predictive factors for node metastasis in patients with clinical stage I non-small cell lung cancer. The Annals of thoracic surgery.

[11] Dranoff G (2004). Cytokines in cancer pathogenesis and cancer therapy. Nature reviews Cancer.

[12] Brunetti G, Bossi A, Baiardi P, Jedrychowska I, Pozzi U, Bacchella L, Bernardo G (1999). Soluble interleukin 2 receptor (sIL2R) in monitoring advanced lung cancer during chemotherapy. Lung cancer.

[13] Brattstrom D, Bergqvist M, Hesselius P, Larsson A, Wagenius G, Brodin O (2004). Serum VEGF and bFGF adds prognostic information in patients with normal platelet counts when sampled before, during and after treatment for locally advanced non-small cell lung cancer. Lung cancer.

[14] Rutkowski P, Kaminska J, Kowalska M, Ruka W, Steffen J (2003). Cytokine and cytokine receptor serum levels in adult bone sarcoma patients: correlations with local tumor extent and prognosis. Journal of surgical oncology.

[15] Raziuddin S, Sheikha A, Abu-Eshy S, al-Janadi M (1994). Circulating levels of cytokines and soluble cytokine receptors in various T-cell malignancies. Cancer.

[16] De Vita F, Orditura M, Galizia G, Romano C, Roscigno A, Lieto E, Catalano G (2000). Serum interleukin-10 levels as a prognostic factor in advanced non-small cell lung cancer patients. Chest.

[17] Martin F, Santolaria F, Batista N, Milena A, Gonzalez-Reimers E, Brito MJ, Oramas J (1999). Cytokine levels (IL-6 and IFN-gamma), acute phase response and nutritional status as prognostic factors in lung cancer. Cytokine.

[18] Kaminska J, Kowalska MM, Nowacki MP, Chwalinski MG, Rysinska A, Fuksiewicz M (2000). CRP, TNF-alpha, IL-1ra, IL-6, IL-8 and IL-10 in blood serum of colorectal cancer patients. Pathology oncology research.

[19] Kaminska J, Nowacki MP, Kowalska M, Rysinska A, Chwalinski M, Fuksiewicz M, Michalski W, Chechlinska M (2005). Clinical significance of serum cytokine measurements in untreated colorectal cancer patients: soluble tumor necrosis factor receptor type I--an independent prognostic factor. Tumour biology.

[20] Bates SE (1991). Clinical applications of serum tumor markers. Annals of internal medicine.

[21] Bodenmuller H, Ofenloch-Hahnle B, Lane EB, Dessauer A, Bottger V, Donie F (1994). Lung cancer-associated keratin 19 fragments: development and biochemical characterisation of the new serum assay Enzymun-Test CYFRA 21-1. The International journal of biological markers.

[22] Bodenmuller H (1995). The biochemistry of CYFRA 21-1 and other cytokeratin-tests. Scandinavian journal of clinical and laboratory investigation Supplementum.

[23] Broers JL, Ramaekers FC, Rot MK, Oostendorp T, Huysmans A, van Muijen GN, Wagenaar SS, Vooijs GP (1988). Cytokeratins in different types of human lung cancer as monitored by chain-specific monoclonal antibodies. Cancer research.

[24] Kosacka M, Jankowska R (2009). Comparison of cytokeratin 19 expression in tumor tissue and serum CYFRA 21-1 levels in non-small cell lung cancer. Polskie Archiwum Medycyny Wewnetrznej.

[25] Ebert W, Hoppe M, Muley T, Drings P (1997). Monitoring of therapy in inoperable lung cancer patients by measurement of CYFRA 21-1, TPA- TP CEA, and NSE. Anticancer research.

[26] Stieber P, Hasholzner U, Bodenmuller H, Nagel D, Sunder-Plassmann L, Dienemann H, Meier W, Fateh-Moghadam A (1993). CYFRA 21-1. A new marker in lung cancer. Cancer.

[27] Pujol JL, Grenier J, Parrat E, Lehmann M, Lafontaine T, Quantin X, Michel FB (1996). Cytokeratins as serum markers in lung cancer: a comparison of CYFRA 21-1 and TPS. American journal of respiratory and critical care medicine.

[28] Takei Y, Minato K, Tsuchiya S, Takise A, Nakano H, Ezawa K, Fueki N, Hoshino H, Naruse I, Nomoto T, Makimoto T, Ishihara S, Saito R, Mori M (1997). CYFRA 21-1: an indicator of survival and therapeutic effect in lung cancer. Oncology.

[29] Hirashima T, Takada M, Komiya T, Nitta T, Masashi K, Masuda N, Matui K, Kikui M, Yasumitsu T, Kawase I (1998). Prognostic significance of CYFRA 21-1 in non-small cell lung cancer. Anticancer research.

[30] Foa P, Fornier M, Miceli R, Seregni E, Santambrogio L, Nosotti M, Cataldo I, Sala M, Caldiera S, Bombardieri E (1999). Tumour markers CEA, NSE, SCC, TPA and CYFRA 21.1 in resectable non-small cell lung cancer. Anticancer research.

[31] Kashiwabara K, Nakamura H, Esaki T (2000). Prognosis in bronchogenic squamous cell carcinoma groups divided according to serum squamous cell carcinoma-related antigen and cytokeratin 19 fragment levels. Clinica chimica acta.

[32] Buccheri G, Torchio P, Ferrigno D (2003). Clinical equivalence of two cytokeratin markers in mon-small cell lung cancer: a study of tissue polypeptide antigen and cytokeratin 19 fragments. Chest.

[33] Niklinski J, Burzykowski T, Niklinska W, Laudanski J, Chyczewski L, Rapellino M, Furman M (1998). Preoperative CYFRA 21-1 level as a prognostic indicator in resected nonsmall cell lung cancer. The European respiratory journal.

[34] Suzuki H, Ishikawa S, Satoh H, Ishikawa H, Sakai M, Yamamoto T, Onizuka M, Sakakibara Y (2007). Preoperative CYFRA 21-1 levels as a prognostic factor in c-stage I non-small cell lung cancer. European journal of cardio-thoracic surgery.

[35] Jung M, Kim SH, Lee YJ, Hong S, Kang YA, Kim SK, Chang J, Rha SY, Kim JH, Kim DJ, Cho BC (2011). Prognostic and predictive value of CEA and CYFRA 21-1 levels in advanced non-small cell lung cancer patients treated with gefitinib or erlotinib. Experimental and therapeutic medicine.

[36] Edelman MJ, Hodgson L, Rosenblatt PY, Christenson RH, Vokes EE, Wang X, Kratzke R (2012). CYFRA 21-1 as a prognostic and predictive marker in advanced non-small-cell lung cancer in a prospective trial: CALGB 150304. Journal of thoracic oncology.

[37] Park SY, Lee JG, Kim J, Park Y, Lee SK, Bae MK, Lee CY, Kim DJ, Chung KY (2013). Preoperative serum CYFRA 21-1 level as a prognostic factor in surgically treated adenocarcinoma of lung. Lung cancer.

[38] Lai RS, Hsu HK, Lu JY, Ger LP, Lai NS (1996). CYFRA 21-1 enzyme-linked immunosorbent assay. Evaluation as a tumor marker in non-small cell lung cancer. Chest.

[39] Kao CH, Hsieh JF, Ho YJ, Tsai SC, Lee JK (1999). Cytokeratin fragment 19 (CYFRA 21-1) in healthy smokers. Anticancer research.

[40] Niklinski J, Furman M, Burzykowski T, Chyczewski L, Laudanski J, Chyczewska E, Rapellino M (1996). Preoperative CYFRA 21-1 level as a prognostic indicator in resected primary squamous cell lung cancer. British journal of cancer.

[41] Yeh JJ, Liu FY, Hsu WH, Wang JJ, Ho ST, Kao A (2002). Monitoring cytokeratin fragment 19 (CYFRA 21-1) serum levels for early prediction of recurrence of adenocarcinoma and squamous cell carcinoma in the lung after surgical resection. Lung.

[42] Muley T, Dienemann H, Ebert W (2003). Increased CYFRA 21-1 and CEA levels are negative predictors of outcome in p-stage I NSCLC. Anticancer research.

[43] Barlesi F, Gimenez C, Torre JP, Doddoli C, Mancini J, Greillier L, Roux F, Kleisbauer JP (2004). Prognostic value of combination of Cyfra 21-1, CEA and NSE in patients with advanced non-small cell lung cancer. Respiratory medicine.

[44] Merle P, Janicot H, Filaire M, Roux D, Bailly C, Vincent C, Gachon F, Tchirkov A, Kwiatkowski F, Naame A, Escande G, Caillaud D, Verrelle P (2004). Early CYFRA 21-1 variation predicts tumor response to chemotherapy and survival in locally advanced non-small cell lung cancer patients. The International journal of biological markers.

[45] Holdenrieder S, Stieber P, VONP J, Raith H, Nagel D, Feldmann K, Seidel D (2006). Early and specific prediction of the therapeutic efficacy in non-small cell lung cancer patients by nucleosomal DNA and cytokeratin-19 fragments. Annals of the New York Academy of Sciences.

[46] Hatzakis KD, Froudarakis ME, Bouros D, Tzanakis N, Karkavitsas N, Siafakas NM (2002). Prognostic value of serum tumor markers in patients with lung cancer. Respiration.

[47] Lee JH, Chang JH (2005). Diagnostic utility of serum and pleural fluid carcinoembryonic antigen, neuron-specific enolase, and cytokeratin 19 fragments in patients with effusions from primary lung cancer. Chest.

[48] Hillas G, Moschos C, Dimakou K, Vlastos F, Avgeropoulou S, Christakopoulou I, Rasidakis A, Bakakos P (2008). Carcinoembryonic antigen, neuron-specific enolase and cytokeratin fragment 19 (CYFRA 21-1) levels in induced sputum of lung cancer patients. Scandinavian journal of clinical and laboratory investigation.

[49] Xu Y, Xu L, Qiu M, Wang J, Zhou Q, Xu L, Wang J, Yin R (2015). Prognostic value of serum cytokeratin 19 fragments (Cyfra 21-1) in patients with non-small cell lung cancer. Scientific reports.

[50] Larsson SC, Orsini N, Wolk A (2010). Vitamin B6 and risk of colorectal cancer: a meta-analysis of prospective studies. Jama.

[51] Abraham NS, Byrne CJ, Young JM, Solomon MJ (2010). Meta-analysis of well-designed nonrandomized comparative studies of surgical procedures is as good as randomized controlled trials. Journal of clinical epidemiology.

[52] Deo SV, Dunlay SM, Shah IK, Altarabsheh SE, Erwin PJ, Boilson BA, Park SJ, Joyce LD (2013). Dual anti-platelet therapy after coronary artery bypass grafting: is there any benefit? A systematic review and meta-analysis. Journal of cardiac surgery.

[53] Moher D, Liberati A, Tetzlaff J, Altman DG (2010). Preferred reporting items for systematic reviews and meta-analyses: the PRISMA statement. International journal of surgery (London, England).

